# Supplementary material for: In Silico Maturation of a Nanomolar Antibody against the Human CXCR2
Source: Biomolecules. 2022 Sep 13;12(9):1285. doi: 10.3390/biom12091285 (PMC9496334; doi:10.3390/biom12091285)
Supplement: Supplementary file 1 [file biomolecules-12-01285-s001.zip › biomolecules-1844565-supplementary.pdf]

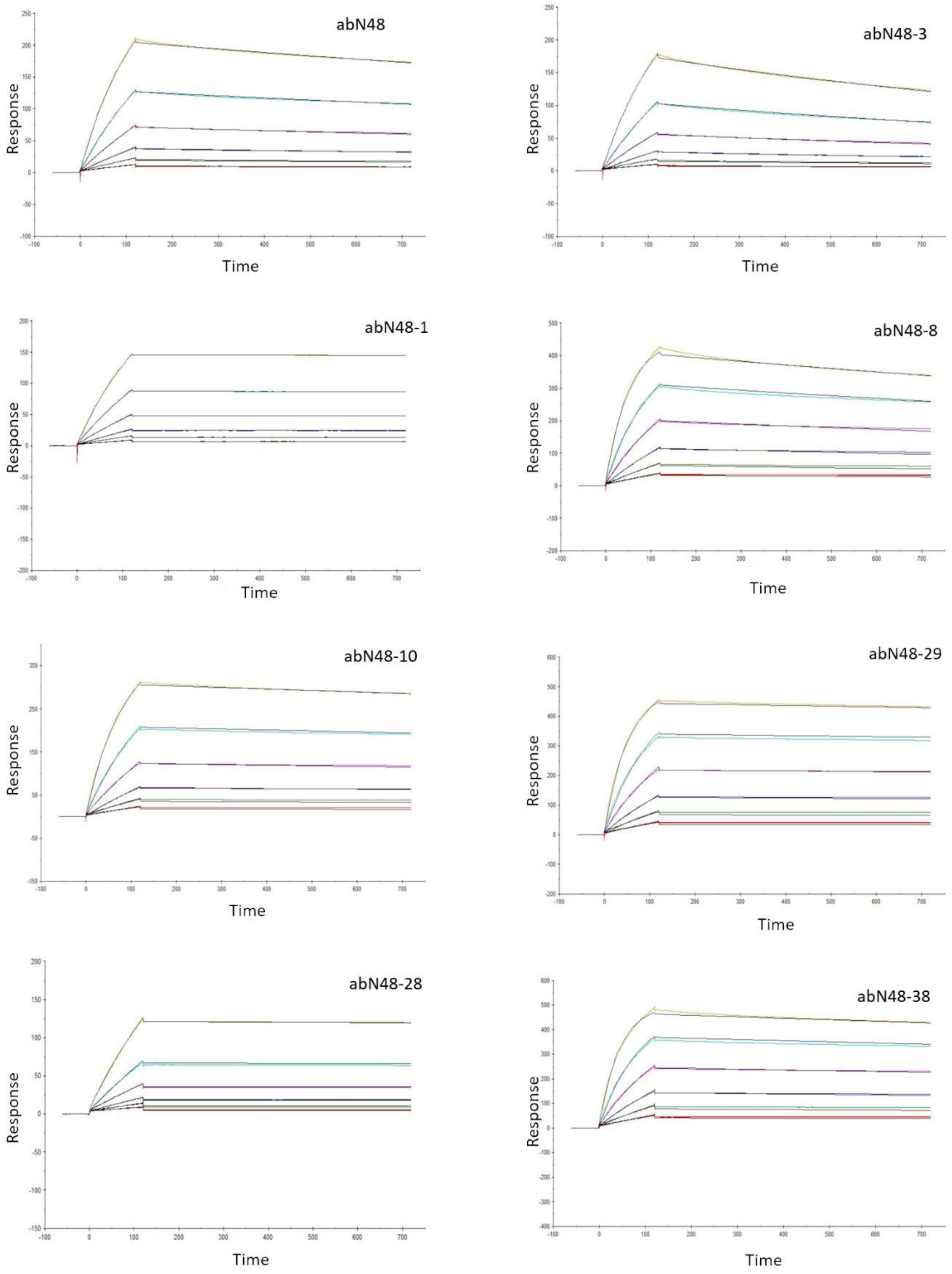

Figure S1: Sensorgrams of abN48 and the affinity maturated antibodies binding to and disassociation from human CXCR2 pepN48 peptide, measured by Biacore T200. Lines of different colors correspond to raw data obtained for different antibody concentrations (red curve: 0.2 nM, green curve: 0.5 nM, blue curve: 1 nM, magenta curve: 2 nM, cyan curve: 5 nM, yellow curve: 10

nM), while the black lines correspond to the best global fit). Kinetics and affinity shown in Figure 1 and Table 2 were calculated from these results.

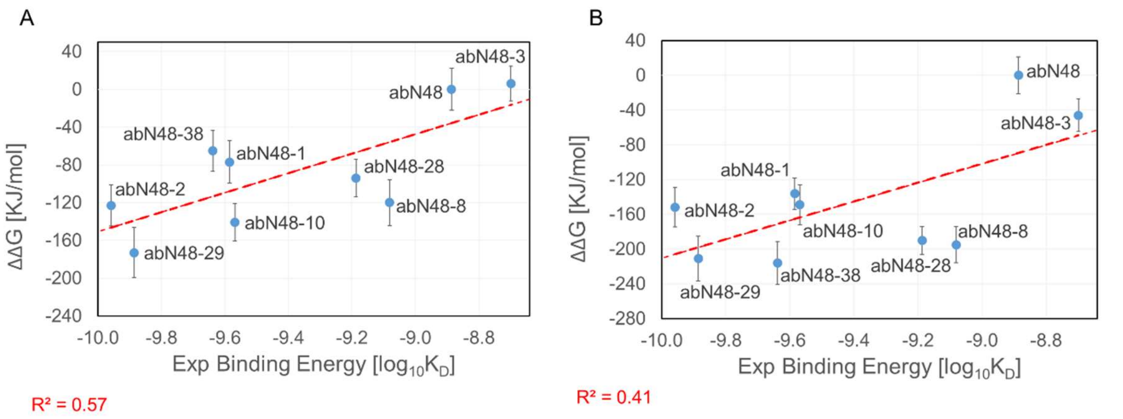

Figure S2: Comparison between experimental binding affinities (Figure S1) and binding free energies computed from configurations sampled according to two different schemes: (A) 10 replicas of 2 ns MD simulations are performed and configurations are extracted every 10 ps from the second half of each replica; (B) a single 25 ns MD simulation is performed and configurations are extracted every 10 ps from the last 20 ns of the trajectory. Both sampling schemes show a fair correlation with experimental data, with scheme A being slightly better.

	CDR-3 Sequence										
	**100**	**101**	**102**	**103**	**104**	**105**	**106**	**107**	**108**	**109**	**110**
abN48	GLY	TYR	CYS	SER	SER	THR	SER	CYS	TYR	ASP	TYR
abN48-1	GLY	PHE	CYS	THR	ARG	THR	ILE	CYS	PHE	VAL	TYR
abN48-2	GLY	TYR	CYS	SER	ARG	THR	ARG	CYS	TYR	ASP	TYR
abN48-3	GLY	TYR	CYS	SER	PHE	ALA	ILE	CYS	PHE	ASP	SER
abN48-8	GLY	TYR	CYS	ASN	ARG	THR	ARG	CYS	TYR	ASP	HIS
abN48-10	GLY	TYR	CYS	SER	ARG	PHE	ASN	CYS	LYS	ASP	TYR
abN48-28	GLY	TYR	CYS	SER	PRO	SER	GLY	CYS	TYR	VAL	TYR
abN48-29	GLY	TYR	CYS	GLY	ARG	ALA	ARG	CYS	THR	SER	PHE
abN48-38	GLY	TYR	CYS	SER	ARG	SER	ARG	CYS	TYR	ASP	TYR

Table S1: Sequences of the antibody used for data in Figure 1
